# Human-exoskeleton control simulation, kinetic and kinematic modeling and parameters extraction

**DOI:** 10.1016/j.mex.2019.08.014

**Published:** 2019-08-23

**Authors:** Maryam Khamar, Mehdi Edrisi, Mohsen Zahiri

**Affiliations:** Electrical Dept., Faculty of Engineering, University of Isfahan, Isfahan, Iran

**Keywords:** Human-exoskeleton system modeling and parameters extraction, Exoskeleton, OpenSim-Matlab interface, Parameter identification

## Abstract

Exoskeletons are new robotic systems that are in close contact with the human body. Thus, their performances are influenced by many factors, including the selection of its structure, actuators, measurement devices, parameters, and mechanism of coupling to the human body. The latter offers numerous challenges to its design, evaluation and modification, including analyzing the effectiveness of the exoskeleton, finding the optimal force for actuators and, discovering the effect of changes in design parameters on human muscle behavior, which are very difficult to measure. Therefore, numerical simulations play an important role in solving these challenges and have the potential to improve treatment strategies and medical decision-making. In this study, a simulation-based method is presented for the designing and analysis of the parameters of an exoskeleton and its wearer’s kinetics and kinematics. Model-based design software, including OpenSim and Inventor, and mathematical software, such as MATLAB, are integrated. This method can assist in the modification of exoskeleton devices and allow physiologists, neuroscientists, and physical therapists to generate new solutions for rehabilitation programs using exoskeletons.

• Using the movements parameters of each individual subject in her/his exoskeleton design.
• Combining the power of OpenSim body movement and the ability of Matlab in mathematical calculations.
• Considering the effect of exoskeleton parameters on each muscle-skeleton movement.

## Graphical abstract

**Figure:**
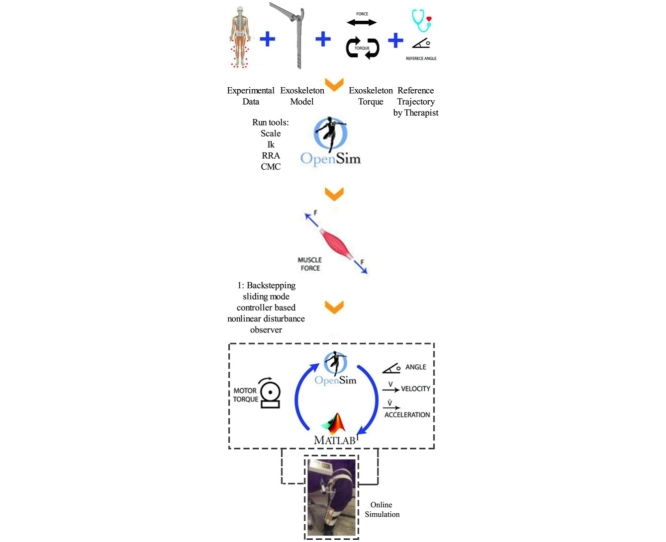

**Specifications Table**

**Table:** 

Subject Area:	Engineering
More specific subject area:	Wearable robots and exoskeleton
Method name:	Human-exoskeleton system modeling and parameters extraction
Name and reference of original method:	Stanev, D. Extendable OpenSim-Matlab Infrastructure Using Class Oriented Mex Interface for C++. 2015; Available from: https://simtk.org/projects/opensimmatlab
Resource availability:	Data, software, movie

## Method details

As exoskeletons are used in human rehabilitation, there are many challenges in the development of exoskeletons in terms of the connection their structure to the human body. These challenges are affected by many factors such as interaction of the exoskeleton and the human body, and exoskeleton actuators way of assisting muscles. Moreover, every human has her/his own movement and force pattern; thus, how experimental data can be extracted from each human body is another challenging issue. This study attempts to tackle some of the challenges in developing an exoskeleton device using simulation methods and software. Simulation can help researchers to develop exoskeleton structures and can give them insight into how this device can help muscle movement. Therefore, a simulation-based method is presented to design and analyze the parameters of an exoskeleton and its wearer’s kinetics and kinematics.

The proposed method consists of four stages. In the first stage, the human model is edited to create a human-exoskeleton model. In the next stage, OpenSim is integrated with MATLAB [1] in order to simulate the effect of exoskeletons on the human body. Considering the sole effect of an exoskeleton on each individual subject with his/her specific characteristics, stages three and four are conducted. In the third stage, the experimental data of a subject is produced by motion capture systems. The computed muscle control (CMC) tool of the OpenSim has been applied to obtain the muscle control values [2]. In stage four, the closed loop simulation of a human-exoskeleton is performed using the results of the third stage. In addition, in this stage, the controller is implemented and simulated to obtain control parameters for the exoskeleton actuators.

## Procedure for simulation

### Stage 1: build an human-exoskeleton model

Step 1: Building an exoskeleton model

The mechanical parts of the exoskeleton are sketched in inventor software [3]. Then, this model should be exported to a file in “.stl” format. These parts of the model should be added to the geometry of the human body in OpenSim model [4].

Step 2: Add the exoskeleton model to the human body model in OpenSim

A set of rigid bodies that represents the exoskeleton system is added to the OpenSim human body model by editing a human-based model file from the OpenSim Documentation [5]. In the **<BodySet>** section of the human body model, we should add the group of exoskeleton bodies including the name, mass properties, and those visible objects associated with each body.

**Figure:**
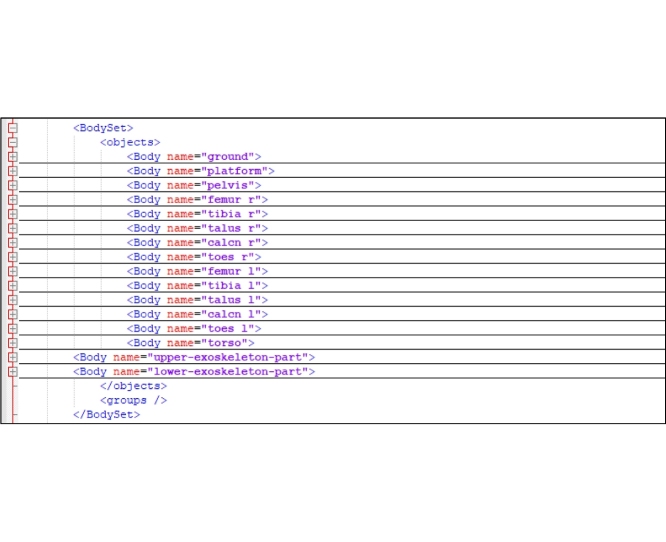

After adding the set of rigid exoskeleton bodies, the relationship between these bodies (i.e., joint definitions) should be defined. A joint defines the kinematic relationship between two frames attached to a child body (B) and parent body (P).

Available joint types in OpenSim model are [5]:

• WeldJoint: introduces no coordinates (degrees of freedom) and fuses bodies together
• PinJoint: one coordinate about the common Z-axis of parent and child joint frames
• SliderJoint: one coordinate along the common X-axis of parent and child joint frames
• BallJoint: three rotational coordinates that are about X, Y, and Z of B in P
• EllipsoidJoint: three rotational coordinates that are about X, Y, and Z of B in P with coupled translations such that B traces and ellipsoid centered at P
• FreeJoint: six coordinates with 3 rotational (like the ball) and 3 translations of B in P
• CustomJoint: user specified 1–6 coordinates and user defined spatial transform to locate B with respect to P

According to the exoskeleton joint type, one of the above joint types is selected in order to define the joint between two rigid bodies of the exoskeleton.

**Fig. 1:**
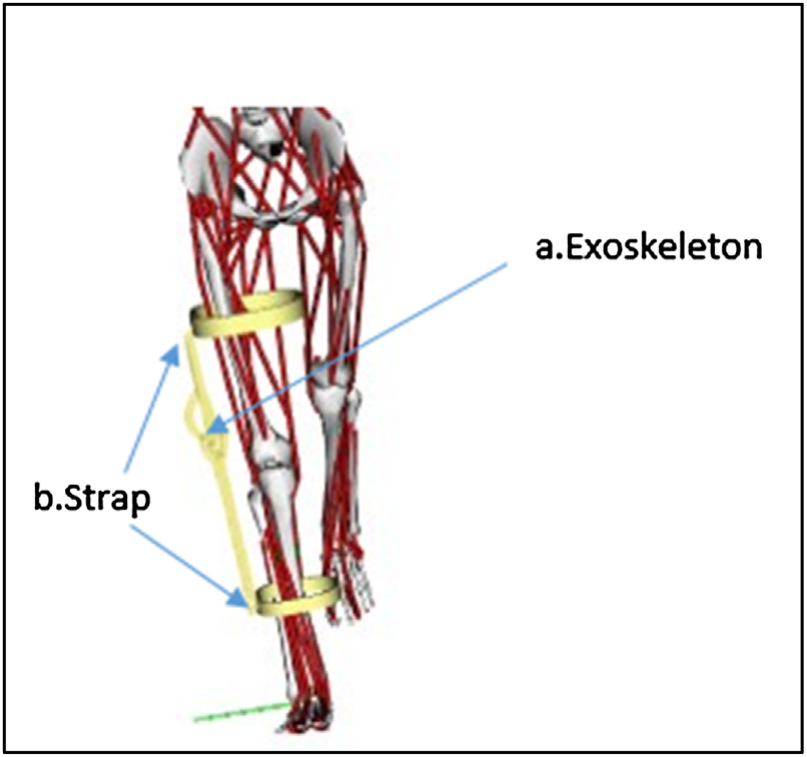
Human–exoskeleton model: a. exoskeleton joint (pin type) b. strap (weldjoint type).

The exoskeleton is secured to the limb by the strapping system. To apply pressure to the tendon, these band-like devices are wrapped around the leg below and above the knee. To model the strapping system that connects the exoskeleton to the human body, the WeldJoint type is used. Fig. 1 shows the human-exoskeleton model.

Step 3: Adding an Additional Actuator

In this step, the “ForceSet” term is found in the file and a New Actuator is added to it. A “CoordinateActuator” object name is also added. The coordinate to which the exoskeleton applied force is associated to “CoordinateActuator”. For example, in the knee exoskeleton, the following code is written to add an actuator to it.

**Figure:**
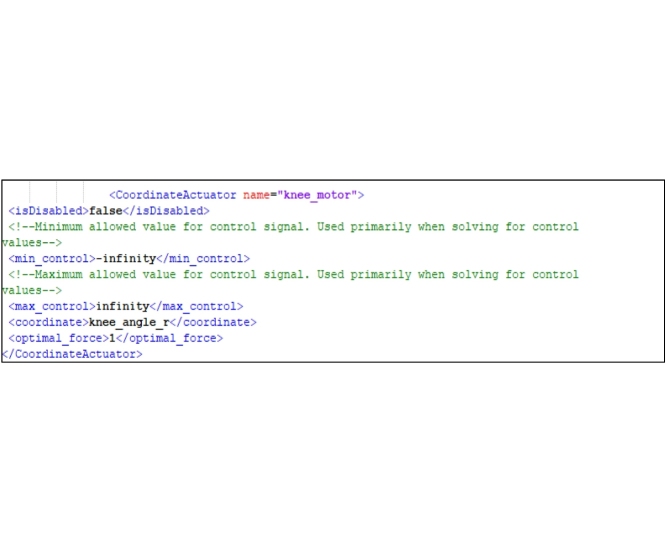

### Stage 2: interface between MATLAB and OpenSim

MATLAB is integrated with OpenSim software with the help of some source files provided by Stanev [6]. This was inspired by a related project conducted in Mokka site [7] in which a user can easily find a file that can interface OpenSim with Simulink. The code that was provided by Mokka [7] is edited in order to provide new capabilities to link a custom exoskeleton to a human model in OpenSim and to harvest both the powerful OpenSim C++ API and MATLAB functionalities. The purpose of this edition is to add an actuator to the model that produces the torque exerted by the exoskeleton.

Step 1: Edit the “EngineMATLAB.h” file provided by Stanev [6] and add the following code to it:

**Figure:**
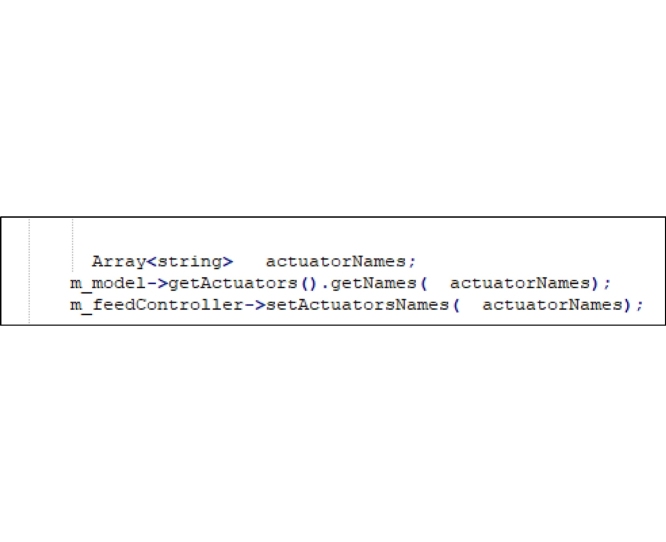

Step 2: Edit “setExcitation” and replace the following code lines in it:

**Figure:**
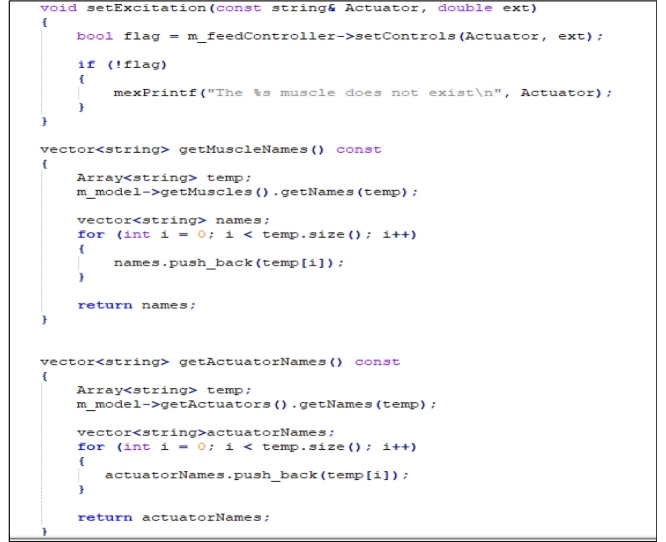

Step 3: Add this code to the “EngineMATLAB.cpp” file provided by Mokka [7]:

**Figure:**
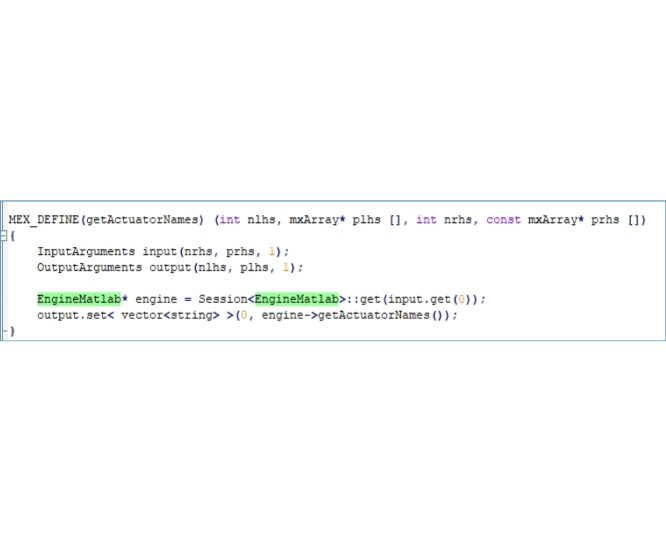

Step 4: Add the following code to the “EngineMATLAB.m” file provided by Stanev [6]:

**Figure:**
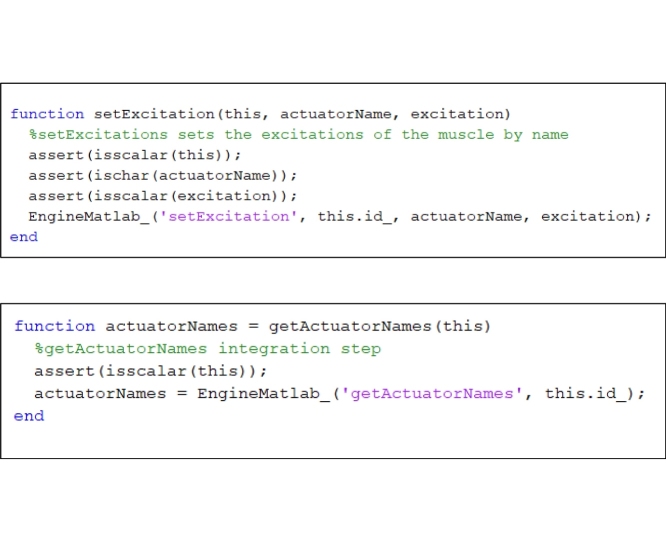

### Stage 3: computation of muscle control of a normal or abnormal gait subject

OpenSim can create dynamic torque-driven simulation of a subject based on his/her integrated 3D motion capture data [2]. In the biomechanics community, C3D is a typical file format for experimental motion capture data. Marker, force plate, EMG, and event data can be stored in the flexible format of C3D. However, OpenSim can analyze “.trc” format data. Thus, the format type of experimental data should be changed to “.trc” using Mokka software. Using the Biomechanical ToolKit, Mokka is a free, open-source, graphical application software [7] that was written for people with non-programming skills to visualize 3D and 2D data in movement science applications like gait analysis, biomechanical research, and sport analysis. Mokka can fully support and open a biomechanics standard C3D file format, and exports its data to commercial formats used in OpenSim.

The first step in the analysis of experimental data in OpenSim is scaling. A model matches a particular subject as closely as possible when the Scale Tool alters the anthropometry of the model. Scaling a model is an iterative process that is typically performed by comparing experimental marker data to virtual markers placed on a model. After running the Scale Tool, OpenSim investigates the "Messages" window for information about the results of scaling, including the overall root mean square (RMS) marker error and the maximum marker error. In general, if the maximum marker error is more than 2 cm for bony landmarks and RMS error more than 1 cm, it visualizes the scaled model’s anatomical marker positions relative to the corresponding experimental markers to see how well the model "fits" the data [5].

Subsequently, the scaling tool is run and minimum error is achieved in order to ascertain that the OpenSim model matches the subject. Then, the inverse kinematic (IK) tool is run. Since forces and moments that produce a motion are not considered in *Kinematics*, mass and inertia properties are not needed when performing IK analyses. The IK tool tries to find the joint angles of the model that best reproduce the experimental kinematics of a particular subject based on *experimental marker* positions. The IK tool computes generalized coordinate values of a pose that "best matches" the experimental marker coordinate values in each time step (frame) of motion as a case of a weighted least squares problem, whose solution aims to minimize the errors between markers and model coordinates. A "Messages" window reports total RMS and maximum marker errors. Therefore, the IK tool runs until the RMS error reaches 2 cm or less [5].

The result of IK can be used in other tools such as the residual reduction algorithm (RRA) to minimize the effects of modeling and marker data processing errors that aggregate and lead to large nonphysical compensatory forces called residuals, and in CMC tools to calculate the muscle force of the subject. Running the RRA tool before the CMC tool is essential [5]. In the simulation procedure, reference trajectory and muscle force generated by the subject are needed.

The outputs of RRA are used in CMC and in gait analysis. One of the RRA outputs is the kinematics of each joint that can be used to investigate and compare the subject’s gait data with normal gait. In this step, a therapist can define a reference trajectory for an exoskeleton. For instance, the therapist can investigate the gait data such as knee motion in the sagittal plane and compare it with normal data. Then, according to the rehabilitation treatment procedure defined for this subject, the reference trajectory can be planned for a knee exoskeleton. Furthermore, the therapist can set the maximum and minimum of flexion and extension of the knee joint, and the speed of motion according to the experimental data and rehabilitation treatment procedure [6].

**Fig. 2:**
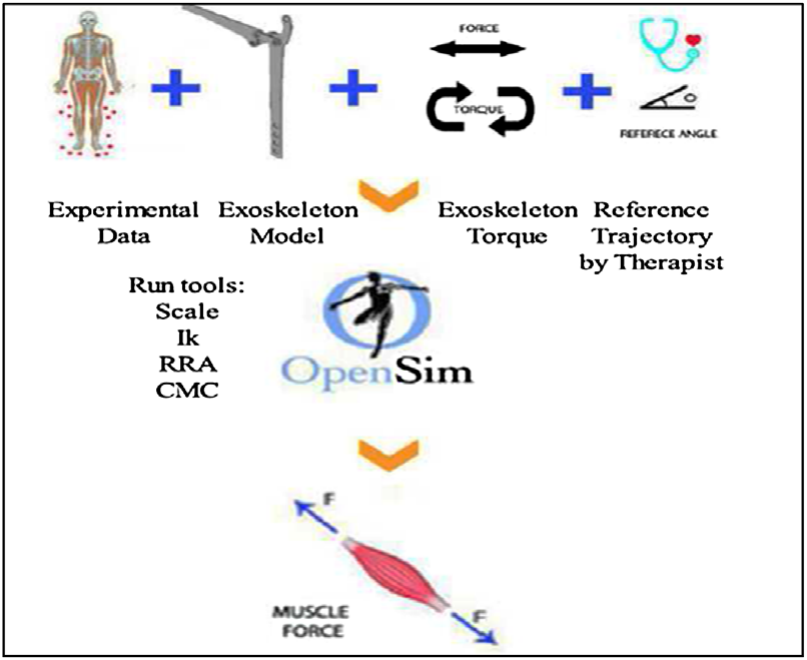
The required data for closed loop simulation of a human-exoskeleton system.

The required data for the simulation procedure consists of the experimental data, exoskeleton model, exoskeleton torque, and reference trajectory. In addition, the muscle force is required to model human force as shown in Fig. 2.

The CMC tool can compute a set of muscle excitations (or more generally, actuator controls) that could drive a dynamic musculoskeletal model to track a set of desired kinematics in the presence of applied external forces (if applicable). The scaled and RRA tools are used for estimating muscle activations and forces during the task using the CMC approach. CMC utilizes a proportional derivative controller to provide kinematics feedback to adjust the model’s position during the simulation, and a combination of feedback control, static optimization, and forward dynamics to estimate muscles’ excitations required for tracking a movement [8].

The CMC was repeated iteratively to achieve the following results:

• Peak errors between experimental data and CMC kinematics of less than 2–5°
• Peak reserve actuator torques of less than 10% of the peak joint torque
• Peak residual forces of less than 10–20 N; peak residual moments of less than 75 Nm (depending on the type of motion)

**Fig. 3:**
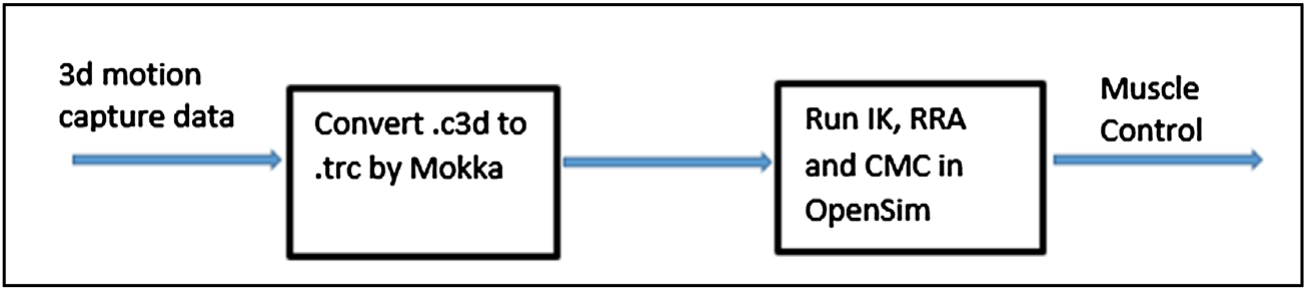
Schematic of calculation of muscle control from motion capture data.

The schematic of this stage is shown in Fig. 3. This stage provides all the input for the simulation procedure. Then, in the next stage, the closed loop simulation can be performed.

Stage 4: Running the simulation of a closed loop control of a human-exosleleton system.

The closed loop system consists of a human and exoskeleton, and the purpose of this system is the tracking of the reference trajectory by the human and exoskeleton system. In the experimental test, the exoskeleton applies force to the human limb and causes flexion or extension of the knee. The force is applied to an extent that the human-exoskeleton system tracks the reference trajectory. However, when using exoskeleton as a wearable robot, sensors can only measure the combined forces of the human-exoskeleton system, thus the exerted force of the human should be estimated by a disturbance observer. Consequently, the output of the CMC is used as an initial values for the forward dynamic tool. It means, the muscle force is not used in controller directly but an estimation of this value with the help of a nonlinear disturbance observer is calculated and used.

**Fig. 4:**
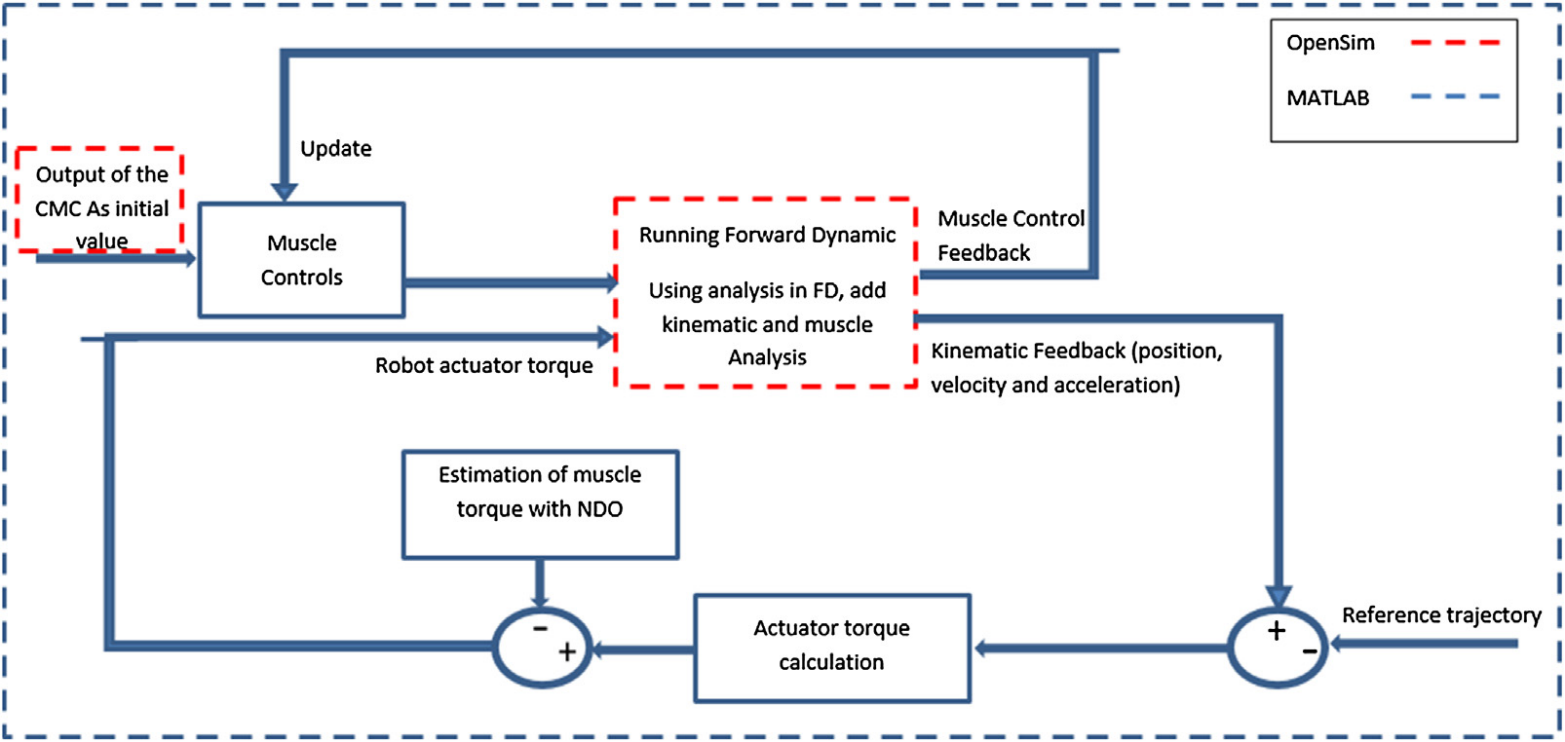
Schematic of online interaction of human and exoskeleton.

To run the closed loop system, a model of a human with all of his/her muscles and bones and an exoskeleton structure should be provided in OpenSim as stated in stage 1. Then, the forces exerted by the exoskeleton to the system should be modeled by editing the OpenSim interface provided by Stanev [6]. The details of necessary modifications are presented in stage 2. Furthermore, the muscle forces should be calculated as in stage 3. These forces model the forces that are applied by the subject when tracking the predefined trajectory. The muscle forces are calculated by using analysis tool when forward dynamic tool is run in OpenSim and are sent back to MATLAB while considering the robot actuator torque is a portion of it. At this stage, all data for running the closed loop simulation are provided. The schematic of this stage is shown in Fig. 4.

Through performing the following steps, the simulation of the exoskeleton interacting with human body is completed.

Step 1: The reference trajectory is implemented. This trajectory is defined by a therapist. The therapist can set the reference trajectories of the human joints freely. However, the therapist can compare trajectories calculated from the captured motion data with normal trajectories, and set new trajectories according to gait data of each subject to achieve a better result in the treatment procedure.

Step 2: The muscle controls, computed using CMC in the third stage, are used in MATLAB to model torque applied by the human subject.

Step 3: The controller presented by Khamar and Edrisi [9] is implemented in MATLAB to produce the actuator control value needed by the exoskeleton to help the human body follow the reference trajectory. This controller has two inputs and three outputs. The inputs of the controller are muscle force (the force generated by the subject’s muscles) and exoskeleton torque in each joint and the outputs are angle, velocity, and acceleration of each joint such as knee, hip, and ankle.

## The parameter extraction procedure

Using the above MATLAB-OpenSim interface and following sections 5.1 and 5.2 of the study by Khamar and Edrisi [9], one can extract the required parameters.

## Method validation

These methods were implemented in the study by Khamar and Edrisi [9] to simulate a knee exoskeleton interaction. First, the human knee exoskeleton model was built. Then, according to the designed procedure, the simulation was performed. After validating the simulation results and setting the controller parameters to reach a specific goal, the experimental test was carried out. The result of the simulation was compared with experimental results. The comparison showed that this method could acceptably simulate a real human-exoskeleton system.

## References

[1] Mansouri M., Reinbolt J.A. (2012). A platform for dynamic simulation and control of movement based on OpenSim and MATLAB. J. Biomech..

[2] Delp S.L., Anderson F.C., Arnold A.S., Loan P., Habib A., John C.T. (2007). OpenSim: open-source software to create and analyze dynamic simulations of movement. IEEE Trans. Biomed. Eng..

[3] INVENTOR. Available from (2018): https://www.autodesk.com/products/inventor/overview.

[4] Ferrati F., Bortoletto R., Pagello E. (2013). Virtual modelling of a real exoskeleton constrained to a human musculoskeletal model. Conference on Biomimetic and Biohybrid Systems.

[5] OpenSim Documentation. Available from (2018): https://simtk-confluence.stanford.edu/display/OpenSim/Documentation.

[6] Stanev D. (2015). Extendable OpenSim-Matlab Infrastructure Using Class Oriented Mex Interface for C++. https://simtk.org/projects/opensimmatlab.

[7] Mokka. Available from (2018): http://biomechanical-toolkit.github.io/mokka/.

[8] Thelen D.G., Anderson F.C., Delp S.L. (2003). Generating dynamic simulations of movement using computed muscle control. J. Biomech..

[9] Khamar M., Edrisi M. (2018). Designing a backstepping sliding mode controller for an assistant human knee exoskeleton based on nonlinear disturbance observer. Mechatronics.

